# P-1231. A Phase 1 Open-label, Parallel-group, Single-dose Study to Evaluate the Pharmacokinetics and Safety of Obeldesivir in Participants with Normal Renal Function and Renal Impairment

**DOI:** 10.1093/ofid/ofaf695.1423

**Published:** 2026-01-11

**Authors:** Elham Amini, Yiannis Koullias, Sharline Madera, NgocQuyen Nguyen, Steve West, Helen Winter, Luzelena Caro

**Affiliations:** Gilead Sciences, Inc., Foster City, CA; Gilead Sciences, Inc., Foster City, CA; Gilead Sciences, Inc., Foster City, CA; Gilead Sciences, Inc., Foster City, CA; Gilead Sciences, Inc., Foster City, CA, USA, Foster City, California; Gilead Sciences, Inc., Foster City, CA; Gilead Sciences, Inc., Foster City, CA

## Abstract

**Background:**

Obeldesivir (ODV) is an ester prodrug of GS-441524 with potential antiviral activity against multiple respiratory viruses. Following oral administration, ODV is extensively hydrolyzed presystemically to GS-441524, which is primarily eliminated through renal clearance. Renal clearance of GS-441524 has previously been characterized following administration of remdesivir, but not ODV.

**Figure:**
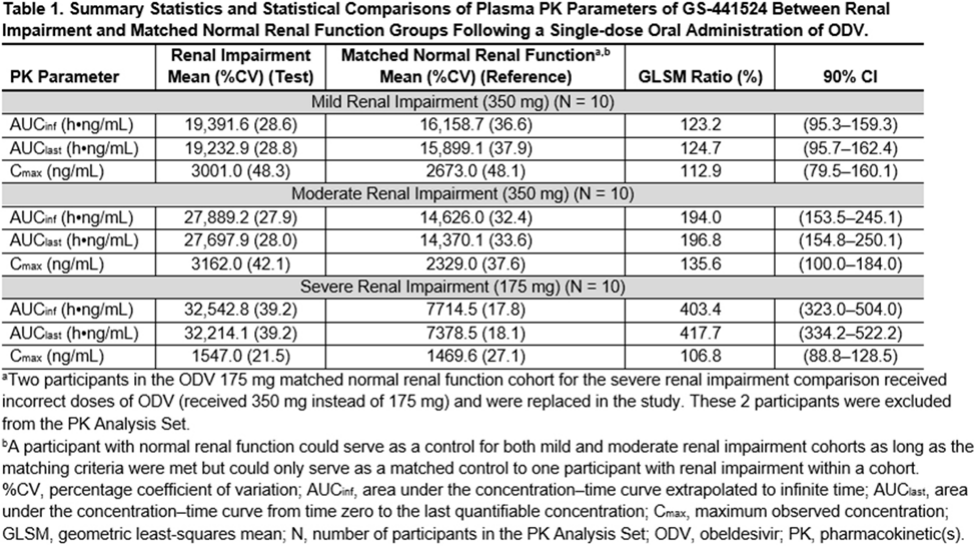

**Figure:**
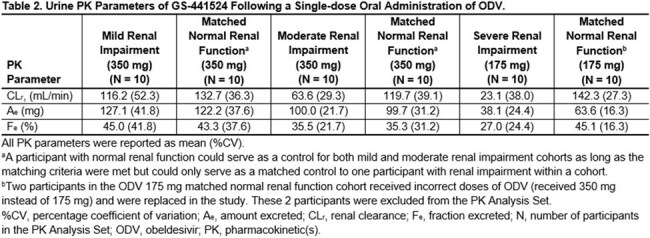

**Methods:**

This was an open-label, Phase 1 study to evaluate the PK, safety, and tolerability of a single dose of ODV in participants with renal impairment (RI) relative to matched control participants with normal renal function. Participants with RI at baseline were enrolled into 3 cohorts based on estimated glomerular filtration rate (eGFR) calculated by the 2021 CKD-EPI creatinine equation: mild RI (60 ≤ eGFR < 90 mL/min/1.73 m^2^), moderate RI (30 ≤ eGFR < 60 mL/min/1.73 m^2^), and severe RI (15 ≤ eGFR < 30 mL/min/1.73 m^2^). Participants with mild or moderate RI and their matched controls received 1 dose of ODV 350 mg and participants with severe RI and their matched controls received 1 dose of ODV 175 mg. Intensive plasma and urine PK sampling occurred relative to the dosing of ODV for up to 120 hours and 72 hours, respectively. PK parameters were estimated for all participants and all renal function groups using Phoenix WinNonlin® software and standard noncompartmental methods. Treatment-emergent adverse events (AEs) and laboratory abnormalities were assessed.

**Results:**

Following single doses of ODV 350 mg or 175 mg, GS-441524 plasma exposures increased approximately 2-fold in moderate RI and approximately 4-fold in severe RI (Table 1). Renal clearance of GS-441524 decreased as baseline renal function decreased (Table 2). All AEs and most laboratory abnormalities were either Grade 1 or 2 in severity among all cohorts. No deaths, AEs leading to discontinuation from study drug or study, or Grade 3 or higher AEs were reported. None of the laboratory abnormalities were considered clinically significant.

**Conclusion:**

Plasma exposures of the ODV circulating metabolite GS-441524 increased as baseline renal function decreased, and renal clearance decreased with greater severity of baseline RI. Single doses of ODV were generally safe and well tolerated in participants with RI and in healthy matched control participants.

**Disclosures:**

Elham Amini, PharmD, PhD, Gilead Sciences, Inc.: Employee|Gilead Sciences, Inc.: Stocks/Bonds (Public Company) Yiannis Koullias, MD, Gilead Sciences, Inc.: Employee|Gilead Sciences, Inc.: Stocks/Bonds (Public Company) Sharline Madera, MD, PhD, Gilead Sciences, Inc.: Employee|Gilead Sciences, Inc.: Stocks/Bonds (Public Company) NgocQuyen Nguyen, PharmD, Gilead Sciences, Inc.: Employee|Gilead Sciences, Inc.: Stocks/Bonds (Public Company) Steve West, MSPH, Gilead Sciences, Inc.: Employee|Gilead Sciences, Inc.: Stocks/Bonds (Public Company) Helen Winter, PhD, Gilead Sciences, Inc.: Former Employee|Gilead Sciences, Inc.: Stocks/Bonds (Public Company) Luzelena Caro, PhD, Gilead Sciences, Inc.: Employee|Gilead Sciences, Inc.: Stocks/Bonds (Public Company)

